# Covid-19-induced pulmonary hypertension in children, and the use of phosphodiesterase-5 inhibitors

**DOI:** 10.12688/f1000research.53966.1

**Published:** 2021-08-11

**Authors:** Herlina Dimiati, Dimas Arya Umara, Iflan Naufal

**Affiliations:** 1 Pediatric, Universitas Syiah Kuala, Banda Aceh, Aceh, Indonesia, Indonesia; 2 Cardiology, Universitas Syiah Kuala, Banda Aceh, Aceh, Indonesia, Indonesia; 3 Family Medicine, Universitas Syiah Kuala, Banda aceh, Aceh, Indonesia, Indonesia

**Keywords:** COVID-19, pulmonary hypertension, Phosphodiesterase-5 inhibitors, Children

## Abstract

Respiratory tract infection caused by severe acute respiratory syndrome coronavirus 2 (SARS-CoV-2) first occurred in Wuhan, China, in December 2019 and was declared as a pandemic by WHO. The interaction between the 2019 coronavirus disease (COVID-19) and pulmonary hypertension (PH) in children is not widely known. Phosphodiesterase-5 inhibitors (PDEI), one class of drugs used to treat PH, including sildenafil, can suppress angiotensin type I (AT-1) receptor expression. Furthermore, it reduces proinflammatory cytokines and infiltrates the alveolar, inhibits endothelial and smooth muscle transition, mesenchymal cells in the pulmonary artery, and prevents clotting and thrombosis complications. Sildenafil has shown positive effects by diverting the blood flow to the lungs in such a way that ventilation is adequate and can also be anti-inflammatory.

## Introduction

Coronavirus disease 2019 (COVID-19) is a respiratory tract infection caused by severe acute respiratory syndrome coronavirus-2 (SARS-CoV-2). In addition, the virus can mutate rapidly and is a zoonotic pathogen that can exist in humans and animals with a diverse range of clinical presentation, from asymptomatic, mild to severe symptoms, and death. ^1^

This respiratory-tract infection, currently declared as a pandemic, was caused by the newly recognized coronavirus emerged in Wuhan, China, in December 2019. ^1, 2^ As of March 19, 2020, there were 209,839 cases with more than 170 countries affected. About 8,778 deaths were reported, with a fatality rate of 4.18%. Furthermore, the incidence in children is lower than adults, and most children, who were confirmed to be positive, contracted the infection from their families. The incidence in children aged 10–19 years was 549 per 72,314 cases or 1% of all cases, while in children aged under 10 years, the incidence was 416 per 72,314 (0.9%) cases. The reported data showed that the mortality rate ranges from 0.3 per 1000 cases in patients under 18 years old. ^3^ Although most people who were confirmed positive had only mild symptoms or were without any complication, about 14% had severe illness needing hospitalization and oxygen support, and 5% needed to be admitted to an intensive care unit. ^1^ In severe cases, infection causes serious acute respiratory disease syndrome (ARDS), sepsis including septic shock, multi-organ failure, including renal failure, or acute heart failure. ^3^ Old age and comorbidities are reported as predictors of death, and D-dimer >1 μg/L when admitted to a health facility is associated with higher case fatality rate. ^4^

The symptoms in children are usually not as severe as adults and generally include cough and fever, which can resolve independently. Furthermore, the signs and symptoms in children are difficult to distinguish from other respiratory diseases. The respiratory tract disease becomes dangerous if it attacks the lungs or causes inflammation of the lungs or pneumonia. The symptoms of pneumonia includes fever, cough, and difficulty in breathing, which is characterized by rapid breathing and shortness of breath. ^5- 7^

SARS-CoV-2 is an enveloped single-stranded RNA virus (ssRNA) and is the seventh strain of coronavirus that can infect humans. This virus is different from other coronaviruses known to cause mild respiratory infections (229E, OC43, NL63, and HKU1). However, it is much the same as the SARS-CoV and the Middle East Respiratory Syndrome Coronavirus (MERS-CoV), which may lead to severe respiratory infections. ^8, 9^ Furthermore, it is suspected that SARS-CoV-2 emerged from bats because it has 89–96% nucleotide similarity to the bat coronavirus. Similar to the case with SARS and MERS, SARS-CoV-2 probably moved from bats to an intermediate host (possibly pangolins, with 91% nucleotide similarity) and was later transmitted to humans. ^10^

The SARS-CoV-2 virus spreads predominantly through droplets produced by sneezing or coughing, and indirectly through infected objects or surfaces. Transmission can be from symptomatic or asymptomatic patients, and the incubation period for the virus is about 2–14 days (5 days on average). Furthermore, the main symptoms experience by the patients includes fever, cough, and dyspnea. Other symptoms include myalgia, anorexia, malaise, painful swallowing, nausea or vomiting, nasal congestion, headaches, and diarrhea. In severe cases, the patient may develop severe pneumonia, ARDS, sepsis, septic shock, and multiple organ dysfunction syndromes (MODS). ^11^ Although the clinical manifestations are predominantly respiratory symptoms, patients may experience severe cardiovascular disorders, including pulmonary hypertension (PH). ^12^

## Pathogenesis of COVID-19

The pathogenesis of SARS-CoV-2 remains unknown, but it is probably not much different from the more widely known SARS-CoV. ^13^ In humans, SARS-CoV-2 primarily infects cells in the airways coating the alveoli. Furthermore, it links to the receptors and enter the cell. The glycoprotein in the envelope spike of the virus binds to the cellular receptor in the form of ACE2 on SARS-CoV-2. Inside cells, the virus clones the genetic material and synthesizes the required proteins, forming new virions appearing on the cell surface. ^14, 15^

In the case of SARS-CoV-2, it is assumed that when the virus enters the cell, the viral RNA genome is discharged into the cell cytoplasm and translated into two polyproteins and structural proteins. Subsequently, the viral genome will begin to replicate. The glycoproteins in the newly formed viral envelope enter the membrane of the endoplasmic reticulum or Golgi cells. Therefore, the formation of the nucleocapsid, composed of RNA genome and nucleocapsid proteins, occurs. The virus particles grow into the endoplasmic reticulum and Golgi apparatus. Finally, the vesicles with virus particles bind to the the plasma membrane and generate new viral components. ^16- 18^

In SARS-CoV, it has been reported that the S protein is the main predictor of virus penetration into host cells. Furthermore, its entry into a cell begins with the fusion of the viral membrane and the plasma membrane of the cell. ^16, 18^ In this process, the S2' protein has a vital role in the proteolytic cleavage process, which mediates the membrane fusion process. In addition to membrane fusion, there is clathrin-dependent and clathrin-independent endocytosis, mediating SARS-CoV into host cells. ^18^

Virus and host factors contribute to infection. The infection severity is determined by the cytopathic effects of the virus and its capability to defeat the immune response. Immune system disorders leads to tissue damage in SARS-CoV-2. The inadequate immune response causes viral replication and tissue damage. In contrast, an excessive immune response could result in tissue damage. ^19, 20^

The immune response induced by SARS-CoV-2 is also not fully known, but can be examined using SARS-CoV mechanisms. When the virus gets into the cell, the viral antigen is presented to antigen presenting cells (APC). The presentation of viral antigens mainly depends on the major histocompatibility complex (MHC) class I molecules. However, MHC class II also plays a role. Antigen presentation also stimulates humoral and cellular immune response mediated by virus-specific T and B cells. In the humoral immune response, IgM and IgG are formed against SARS-CoV. IgM to SARS-CoV disappears by the end of week 12, but IgG can last long. ^13, 19, 20^

Viruses have mechanisms to evade the host immune response. SARS-CoV may produce double-membrane vesicles without pattern recognition receptors (PRRs) and duplicate in these vesicles in such a way that the host cannot recognize them, and can also inhibited the IFN-I line. ^13, 19^

The role of ACE2in COVID-19 has been extensively discussed and researched because SARS-COV-2 uses this transmembrane receptor to cross cell membranes, and its expression is positively associated with infectivity. The enzyme that converts ACE and ACE2 has a balancing effect and leads to pulmonary and systemic effusions. ACE converts angiotensin to angiotensin-II *via* activation of the angiotensin-II type 1 receptor pathway, causing a proliferative response and prothrombotic vasoconstriction. In the other hand, ACE2 converts angiotensin-II to angiotensin (1–7) heptapeptide, which reverses angiotensin-II action with its antithrombotic effects and antiproliferative vasodilator. Subsequently diminishing the part of ACE2 in COVID-19 seriousness because of its suggestions for viral infectivity, which can be excessively shortsighted, as this receptor has been demonstrated to be engaged with the pathogenesis of the different instruments causing lung and cardiovascular harm. Moreover, ACE2 has an endothelial assurance impact by turning around the impacts of ACE angiotensin II. Studies have revealed that ACE2 overexpression decline atherosclerosis by protecting endothelial capacity and lessening the incendiary reaction through diminished articulation of MCP-1, VCAM-1, and E-selectin brought about by angiotensin II. This endothelial defensive impact is the reason for the antithrombotic impact. Therefore, ACE2 expression and thrombotic load in patients with pulmonary embolism has been proven to be oppositely correlated. ^21- 24^

Despite the fact that angiotensin II is the primary substrate of ACE2, it can also degrade angiotensin I into angiotensin and play a role in peptide hydrolysis. The ACE2/angiotensin axis plays a role in protective physiological mechanisms against the activation of classic renin angiotensin system (RAS). ^8, 21- 24^

ACE2 not only plays a crucial role in the cardiovascular system, but also serves as a receptor for the coronavirus, which binds directly to the virus surface peak protein. SARS-CoV-2 enters the cells through the ACE2 receptor, which is mostly found in the lugs (especially in alveolar type II cells). ACE2 is also present in large quantities in the heart. In addition, ACE2 was also reported to be found on intestinal epithelium, vascular endothelium, and kidney;this is the basis for multi-organ dysfunction mechanism that occur in SARS-CoV-2. ^8, 12^

Infection of SARS-CoV-2 begins when the viral surface spike protein bound to the ACE2 receptor after being activated by transmembrane protease serine 2 (TMPRSS2). ^12^ The unification of the viral spike protein and ACE2 receptor will downregulation the activity on the cell surface. it can cause the enzyme protective effect disappeared. The re-aggregation of ACE2 in the lungs can facilitate infiltration of neutrophil responding towards endotoxins and lead to uncontrolled accumulation of angiotensin II and excessive local activation of RAS, which can lead to lung and myocardial damage. ^8, 12, 22- 25^

The increased oxygen demand due to systemic infections and hypoxia from COVID-19 can cause an imbalance between oxygen demand and supply, which will lead to myocardial injury. The mechanism of myocardial injury due to Covid-19 is not fully recognized, and myocardial traces may result from viral myocarditis and/or an ischemic process. Previous studies indicate that in35% of patients with Covid-19, the genome is identified in the heart, raising the suspicion that the virus can directly infect the myocardium and cause myocarditis and myocardial damages. SARS-CoV-2 can have a similar mechanism as SARS-CoV because they have homologous genomes. ^10, 12^

In inflammatory responses, ACE2 is involved in regulating the innate immunity, and its expression reduces the release of cytokines. The protein spike of SARS-CoV binding to ACE2 will reduce the regulation and develop lungs injury by activating the angiotensin II type 1 receptor by angiotensin II. ^22^ ACE2 expression modification on the surface of pneumocytes is dynamic and vital to oversee inflammation of neutrophil during infection. Thus, in the first stages of infection, diminished ACE2 expression would not affect development of the inflammatory response into deactivated aggression. Latent recovery of ACE2 will help reduce inflammatory infiltrate, preventing excessive injury. ^23^ Therefore, the protein spike of SARS-CoV binds to ACE2 to get into cells and decreases surface ACE2 expression either by internalization or linking to endogenous ACE2, resulting in reduced ACE2 regulation. ^22- 24^ The reduced ACE2 expression results in diffuse endothelial dysfunction, leading to severe lung impairment concomitant with inflammatory infiltrates and also edema, extensive thrombosis, and homeostasis changes in vascular. ^24, 26^

COVID-19 develops a cytokine storm, an uncontrolled systemic inflammatory response due to the deliverance large amounts of proinflammatory cytokines (IFN-α, IFN-γ, IL-1β, IL-2, IL-6, IL-7, IL-10, IL-12, IL-18, IL-33, TNF-α, TGFβ, CCL2, CCL3, CCL5, CXCL8, CXCL9, and CXCL10), Granulocyte-colony stimulating factor (GCSF), monocyte chemoattractant protein 1, and macrophage inflammatory protein 1α, which contribute to myocardial injury, acute respiratory distress syndrome (ARDS), lungs damage and fibrosis. This results in functional disability, disease severity and dysfunction in various organs. Some research have reported high levels of proinflammatory cytokines in severe COVID-19 patients. ^11, 13, 15^

Systemic hyper-inflammation and cytokine storms can eventually lead to an increase of coagulation cascade activation. The hypercoagulation state will intensify the risk of thrombosis and thromboembolism in both the veins and arteries. ^11, 13, 16^ Furthermore, coagulation disorder often found in patients with severe COVID-19 and usually has a poor prognosis. Increased D-dimer, prolonged prothrombin time (PT) and activated partial thromboplastin time (APTT), and thrombocytopenia are common parameters in coagulopathy. ^27, 28^ Coagulopathy causes hypercoagulation, which can enhance the risk of thrombosis complications as well as venous and arterial thromboembolism. ^11, 13, 16, 17, 19, 20, 27- 29^ Venous thromboembolism is the most frequent complication of thrombosis in severe cases, nearly 27% of patients treated in intensive care units. ^27- 29^ In patients with mild manifestations of COVID-19, proinflammatory chemokines and cytokines, as well as venous thromboembolism do not upgrade, even at the time of symptoms. ^30^

## Pulmonary hypertension (PH) in COVID-19

The COVID-19 and PH incidence in adults was 2.1 cases per 1000 patients. Meanwhile, there are few reports of this in children. Some contributing factors to its incidence with PH in children include the rarity of this disease and non-universal testing. However, drugs for treating PH may may protect children against infection. ^31^

Some putative instruments related with PH in COVID-19 start when the limiting of the ACE2 receptor and protein spike of SARS-CoV-2, bringing about serious provocative responses prompting diffuse pneumonic alveolar harm, apoplexy, endothelitis, angiogenesis and endothelial brokenness. ^31- 37^ Hemodynamic status in patients with ARDS is comparable with expansion in the diastolic pneumonic slope (the contrasts between the pressing factor of diastolic aspiratory corridor and aspiratory supply route wedge) >7 and pneumonic vascular obstruction (PVR) >3 Wood units. This is regular for this pre-capillary PH type. ^38, 39^ The continuous examination investigates the advantage of specific treatments for PH, for instance ambrisentan, sildenafil, iloprost, iNO, recombinant ACE2, vasoactive gut peptide analogs (VIP), and tocilizumab in COVID-19. ^41- 42^

PH is the increase of the mean pulmonary arterial pressure (PAPm) ≥ 25 mmHg at rest, or ≥30 mmHg during activity compared with normal PAPm (<15 mmHg) and PVR index >3 units of Wood units measured using right heart catheterization. PH is a pathological condition that can complicate most cardiovascular and respiratory system diseases. ^43^

## Use of sildenafil to manage PH in children with COVID-19

During the pandemic, each child with a history of PH or newly diagnosed PH must be examined for Covid-19 when symptoms occur (fever, respiratory distress, or hypoxemia). If an antigen test shows a negative results, nevertheless the clinical COVID-19 is suspected, an antibody screening must be conducted. ^31^ High-resolution CT (HRCT) capable for measuring changes in pulmonary blood volume and show prominent abnormalities according to "Pruning" of blood vessels and evaluate pulmonary vascular dysfunction. HRCT findings are highly related with increased pulmonary vascular resistance. ^44^

To date, the literature related to PH therapy in children, especially clinical trials, is very limited. Generally, the treatment for PH in children follows the PH management algorithm in adults with some adjustment. ^45^ Current pharmacological therapies used for the treatment of PH include the prostacyclin class (epoprostenol), prostacyclin analogues (beraprost, iloprost, treprostinil), endothelin receptor antagonists (bosentan), nitric oxide (NO), phosphodiesterase-5 inhibitor (sildenafil), and combinations of these preparations. ^46^

The therapy of PH that occurs with COVID-19, in addition to medicine, oxygen should be given if the oxygen saturation <92%. Inhaled nitric oxide (iNO) is well known to treat ARDS and PH. Phosphodiesterase-5 inhibitors (PDEI), especially sildenafil, may reduce angiotensin type I (AT-1) receptors and degrade proinflammatory cytokines and infiltration of inflammatory cell in alveoli. ^31^

There are currently many studies examining the effect of sildenafil for treating the PH in COVID-19, but mainly for adults and only a few reports of cases for children. Sildenafil is a potent inhibitor of phosphodiesterase-5, which can accumulate and increase the activity of cyclic guanosine monophosphate (cGMP) that works synergistically with NO. ^47^ The Food and Drug Administration (FDA) has recommended sildenafil for the treatment of PH since 2005. To date, this medicine is the most frequent medication in pediatric patients with secondary PH. ^48, 49^ Sildenafil is preferred because of the oral packaging in such a way that it is easy to administer. The usual dose of Sildenafil for children is 0.5–1 mg/kg, given 3–4 times a day. Administration of sildenafil in high doses can cause side effects such as erection and systemic hypotension. ^48^

Several pilot studies on the safety of sildenafil have been and are being carried out. In one phase 3 study at Tongji Hospital, the 0.1 g/day was administered for 14 days (in COVID-19 patients between February 9 to November 9, 2020). ^50^ Furthermore, another study conducted in Brazil on children with positive COVID-19, admitted to ICU and receiving nitric oxide and/or sildenafil (0.5–2 mg/kg/dose each 4–6 hours with a maximum of 20 mg/dose every 8 hours) in patients with persistent hypoxemia showed encouraging results. ^51^ A survey conducted to 300 respondents in China, there were 120 respondents from children to adults aged 32.3 +11.5 years with PH. The medications given were bosentan, ambrisentan, tadalafil, and sildenafil. Bosentan and ambrisentan are widely used because they are cheap. There were 69 respondents (57.5%) that received sildenafil and showed good clinical results. ^52^

## Conclusion

There is currently evidence of options for PH management in children. Sildenafil appears to be effective in improving pulmonary vascular hemodynamics. However, it has been shown that no single type of drug has been proven to be better than others. Furthermore, oral sildenafil is an interesting and effective therapy because it is easy to administer, has minimal side effects, and is less expensive than other therapeutic options. Randomized clinical trials with large sample sizes from various research centers are necassary to ensure the safety and optimal dosage of sildenafil in children with COVID-19.

## Data availability

### Underlying data

No data are associated with this article.
