# Supplementary material for: Covid-19-induced pulmonary hypertension in children, and the use of phosphodiesterase-5 inhibitors
Source: F1000Res. 2022 Sep 12;10:792. Originally published 2021 Aug 11. [Version 2] doi: 10.12688/f1000research.53966.2 (PMC11369592; doi:10.12688/f1000research.53966.2)
Supplement: Peer review history

# Reviewer report: Reviewer response for version 2

**DOI:** 10.5256/f1000research.138125.r314866

**Published:** 2024-09-02

**Authors:** Mahrus A Rahman, Prima Nastiti

**Affiliations:** 1 Department of Pediatrics, Universitas Airlangga, Surabaya, East Java, Indonesia

The results of this research is a significant contribution to the literature. I investigated pulmonary arterial hypertension therapy in children.

 The pathogenesis of pulmonary arterial hypertension is involving the role of remodelling that occurs in all layers of the pulmonary arterial wall. There are increasing of proliferative and apoptotic pathway activities, thickening of smooth muscle cell layer, increasing growth of endothelial cells and changes of vasoactive substances homeostasis. TGF-β has a role in proliferation, transformation, apoptosis, deposition of matrix extracellular protein, as profibrotic cytokine and induction of the secretion of proinflammatory cytokine. There is the activity of elastase proteolytic enzyme and matrix metalloproteinase (MMP) in the pulmonary arterial wall that induces the secretion of mitogenic growth factors from extracellular matrix protein. The adventitial fibroblast proliferation is increase beyond the smooth muscle cells proliferation, the dramatic increase in collagen synthesis, fibronectin and tropoelastin mRNA occurs, followed by increased deposition of these proteins. Fibroproliferative changes in the adventitial layer correlate with the narrowing of vascular lumen and progressive decrease in response to the vasodilation.  Tissue inhibitor of metalloproteinase (TIMP) is an endogenous inhibitor of MMP. MMP expression and activity is increased in experimental pulmonary arterial hypertension. Imbalance of MMP-TIMP causes deposition of ECM and fibrosis of pulmonary artery wall.  

 An enzyme homolog known PDE5 contributes to it being easier for cGMP and/or cAMP to break down. Since PDE5 inhibitors restrict this enzyme's activity, systemic and pulmonary pressure are reduced. Furthermore, because pulmonary vascular resistance decreased compared with systemic vascular resistance, PDE5 inhibitors are more selective for pulmonary vasodilators. Since it improves pulmonary pressure, cardiac index, exercise capacity, and functional class—all indicators of the severity of the disease—sildenafil has emerged as the preferred treatment option for pulmonary arteriaI hypertension.

 I propose that the author will continue on conducting the meta-analysis of  pulmonary arterial hypertension treatment especially in COVID 19 infection.

Is the review written in accessible language?

Yes

Are all factual statements correct and adequately supported by citations?

Yes

Are the conclusions drawn appropriate in the context of the current research literature?

Yes

Is the topic of the review discussed comprehensively in the context of the current literature?

Yes

Reviewer Expertise:

Pulmonary Arterial Hypertension in Congenital Heart Disease Therapy

We confirm that we have read this submission and believe that we have an appropriate level of expertise to confirm that it is of an acceptable scientific standard.

# Reviewer report: Reviewer response for version 1

**DOI:** 10.5256/f1000research.57403.r138090

**Published:** 2022-05-26

**Authors:** Ajay Kumar Mishra

**Affiliations:** 1 Department of Cardiovascular Medicine, St Vincent Hospital, Worcester, MA, USA

In this review, the authors have discussed COVID 19 pulmonary hypertension in children and the use of phosphodiesterase-5 inhibitors

 I have the following recommendations:

1. Authors have given many details on COVID 19 per se and much fewer details on pulmonary hypertension, and pulmonary hypertension in children
2. Kindly include all the articles mentioning pulmonary hypertension in children and elaborate on them
3. Kindly include clinical evidence in terms of clinical presentation, diagnosis, management, and outcomes in the paper
4. PMID: 34460150 ^1^, refer, cite, and discuss this retrospective study including pulmonary hypertension in children.
5. PMID: 33525228 ^2^, PMID: 33352654 ^3^,PMID: 32889497 ^4^ refer, cite, and discuss the impact/outcome of pulmonary hypertension in adults and compare with children.
6. Kindly mention in detail the study type, sildenafil dose, form, duration, and impact on outcome in the discussion based on clinical evidence
7. Incorporate international data, if not available please discuss this in limitations

Is the review written in accessible language?

Partly

Are all factual statements correct and adequately supported by citations?

Partly

Are the conclusions drawn appropriate in the context of the current research literature?

No

Is the topic of the review discussed comprehensively in the context of the current literature?

Partly

Reviewer Expertise:

Cardiology and Internal Medicine

I confirm that I have read this submission and believe that I have an appropriate level of expertise to confirm that it is of an acceptable scientific standard, however I have significant reservations, as outlined above.

## References

[1] : Outcomes of COVID-19 infection in pediatric pulmonary hypertension: A single-center experience. *Pediatr Pulmonol* .56(12) : 10.1002/ppul.25650 3960-3965 10.1002/ppul.25650 34460150 PMC8662244

[2] : An Update on Pulmonary Hypertension in Coronavirus Disease-19 (COVID-19). *Acta Biomed* .2020;91(4) : 10.23750/abm.v91i4.10698 10.23750/abm.v91i4.10698 33525228 PMC7927547

[3] : Covid-19 pneumonia and pulmonary vascular disease: A UK Centre perspective. *Respir Med Res* .2020;78(100781) : 10.1016/j.resmer.2020.100781 10.1016/j.resmer.2020.100781 32889497 PMC7398050

[4] : COVID-19 and Pulmonary Hypertension in Children: What Do We Know So Far?. *Medicina (Kaunas)* .2020;56(12) : 10.3390/medicina56120716 10.3390/medicina56120716 33352654 PMC7765781

# Response

**Published:** 2022-09-27

**Authors:** Herlina Dimiati

**Affiliations:** Universitas Syiah Kuala, Indonesia

Dear Prof  Ajay Kumar Mishra

 Thanks for the input regarding the use of ”Phosphodiesterase-5 inhibitors (Sildenafil)” to decreased pulmonary artery pressure in complication of COVID-19. The revision of this paper, we have refined the mechanism of action of the drug, the dose of the drug and many study has been running and is being carried out to assess the effectiveness of the drug in the pediatrics patients. The conclusion of this paper, the role of this drug to decreased pulmonary pressure has been described.

# Reviewer report: Reviewer response for version 1

**DOI:** 10.5256/f1000research.57403.r91674

**Published:** 2021-10-05

**Authors:** Sukman Tulus Putra

**Affiliations:** 1 Department of Child Health, Division of Pediatric Cardiology, Dr. CiptoMangunkusumo Hospital, Faculty of Medicine, University of Indonesia, Jakarta, Indonesia

In the literature review, it was not very clear what the correlation between pulmonary hypertension and the Covid-19 virus is. Particularly the pathomechanism of Covid-19 for the pathogenesis of pulmonary hypertension. So it would be a good point after the discussion of therapy of sildenafil, which is established as a drug of choice for pulmonary artery hypertension if there was an explanation of how the virus can cause pulmonary hypertension based on the literature. 

 In summary, this literature review is quite relevant for the current pandemic situation and it is worth indexing. But it would be better if minor revision to cite the literature regarding the mechanism of pulmonary hypertension that could be happening in Covid-19 infection which could be treated using phosphodiesterase-5 inhibitors.

Is the review written in accessible language?

Yes

Are all factual statements correct and adequately supported by citations?

Yes

Are the conclusions drawn appropriate in the context of the current research literature?

Partly

Is the topic of the review discussed comprehensively in the context of the current literature?

Yes

Reviewer Expertise:

NA

I confirm that I have read this submission and believe that I have an appropriate level of expertise to confirm that it is of an acceptable scientific standard, however I have significant reservations, as outlined above.

# Response

**Published:** 2022-09-27

**Authors:** Herlina Dimiati

**Affiliations:** Universitas Syiah Kuala, Indonesia

Dear Prof Sukman Tulus Putra

 Pulmonary arterial hypertension in COVID-19 cases is associated with several concomitant conditions of acute respiratory distress syndrome (ARDS) and pneumonia which often complicate the course of disease including coagulopathy and thrombosis.

 Various meta-analyses have shown that the rate of thromboembolic complications in COVID-19 patients was 90%.  The effect of ARDS and pneumonia on cardiovascular function is well known and the interconnected anatomical and physiological structures making the pulmonary and right ventricular (RV) circulation most susceptible to disturbances in lung disease both acute and chronic. The incidence of macro and micro pulmonary thrombosis that accompanies COVID-19 pneumonia is believed to further aggravate the hemodynamic problem of the pulmonary circulation through an increase in pulmonary artery pressure.

 Pulmonary hypertension and RV dysfunction have been shown to influence clinical outcomes in COVID-19 patients, but until now in the child group, there is no supporting data.
